# Genomic Biomarkers and Mutational Landscape of Nonsyndromic Hearing Loss (NSHL) in the Singaporean Population: Clinical Translational Implications

**DOI:** 10.3390/biom16030352

**Published:** 2026-02-26

**Authors:** Che Kang Lim, Mei Shuang Cheng, Gerard Low, Joyce Zhi’en Tang, Jia Hui Ng, Ni Gin Ong, Pei Shan Leem, Su Ann Lim, Jiun Fong Thong, Vanessa Yee Jueen Tan

**Affiliations:** 1 Department of Clinical Translation Research, Singapore General Hospital, Singapore 169608, Singapore; 2 Medicine Academic Clinical Programme (ACP), Duke-NUS Medical School, Singapore 169857, Singapore; 3 School of Life Sciences and Chemical Technology, Ngee Ann Polytechnic, Singapore 599489, Singapore; 4 Department of Otorhinolaryngology—Head & Neck Surgery, Singapore General Hospital, Singapore 169608, Singaporeleem.pei.shan@sgh.com.sg (P.S.L.);; 5 Surgery Academic Clinical Programme (ACP), Duke-NUS Medical School, Singapore 169857, Singapore

**Keywords:** nonsyndromic hearing loss, whole-exome sequencing, genetic biomarkers, precision medicine

## Abstract

Nonsyndromic hearing loss (NSHL) is a highly prevalent, genetically heterogeneous condition, yet its molecular basis in the Singaporean population remains underexplored. We performed whole-exome sequencing and integrative bioinformatics analysis in 115 patients with NSHL to define population-specific genetic biomarkers. A molecular diagnosis was achieved in 57% of cases, with 76% of identified variants classified as pathogenic or likely pathogenic and 24% exhibiting high pathogenic potential. Common East Asian NSHL genes, including *GJB2*, *SLC26A4*, and *OTOF*, were frequently detected alongside less prevalent genes such as *ACTG1*, *CEACAM16*, *COL11A2*, *DIAPH1*, *KCQN4*, *MYH14*, *MYO6*, *MYO7A*, *MYO15A*, *SLC17A8*, *SMPX*, *STRC*, *TJP2*, *TMC1*, *TMPRSS3*, highlighting extensive genetic heterogeneity. Notably, multiple novel variants, including *MYO6* c.554-2A>G, and TNC p.N750Y, were identified, expanding the known mutational spectrum of *NSHL*. Genotype–phenotype correlations revealed that *GJB2* variants were primarily associated with mild to moderate hearing loss, whereas *SLC26A4* variants correlated with severe to profound phenotypes in the Singaporean populations. Collectively, our study provides important insights into the genetic architecture of NSHL in Singapore’s population. In addition, it supports improved molecular diagnosis yield and informed clinical management decisions as well as the advancement of precision medicine approaches aimed at reducing the burden of hearing loss in the region.

## 1. Introduction

Hearing loss represents one of the world’s most pervasive sensory impairments, impacting approximately one in five people globally. According to the World Health Organization (WHO), over 1.5 billion people currently live with some degree of hearing loss, and over 5% of the world’s population (430 million people), require rehabilitation to address their disabling hearing loss [1]. This burden is projected to grow substantially, with over 700 million people expected to require hearing rehabilitation by 2050. The unaddressed cost to the global economy is estimated by the WHO at USD 980 billion annually [1]. On a regional level, the challenge is equally pronounced. In Singapore, four out of every 1000 newborns are identified with a significant hearing impairment [2], underscoring the substantial clinical and developmental urgency of early detection and intervention within the local population.

Amongst the myriad of known causes, genetic factors are recognized as the leading known etiology of hearing loss, accounting for approximately 50% of all cases. Approximately 70% of hereditary hearing loss cases are classified as non-syndromic hearing loss (NSHL) [3,4]. Non-syndromic hearing loss (NSHL) is a partial or complete loss of hearing that occurs without any other associated signs or symptoms. One common classification method of NSHL is by the condition’s pattern of inheritance: autosomal dominant (DFNA), autosomal recessive (DFNB), X-linked (DFNX), or mitochondrial (no special designation). Most cases of NSHL are sensorineural, resulting from disruption of essential cellular function in the inner ear. Rarely, cases are described as conductive, resulting from defects in the development of middle ear structures [5].

Early detection and accurate etiological diagnosis are crucial, as untreated hearing impairment can affect language development, education, and social integration [6]. However, there is difficulty in identifying NSHL-associated variants present in individuals due to the highly heterogenous genetic etiology of NSHL. To date, over 150 distinct genes have been identified and the number continues to grow [7,8,9]. In addition, the distribution of NSHL-related genes varies across populations and ethnic groups [10]. This limits the applicability of existing gene panels and leads to complications when interpreting variants of uncertain significance despite the advancement in sequencing technology. While Northeast Asian populations, including Chinese and Japanese cohorts, have been extensively studied [11,12,13], there is a notable lack of Southeast Asian-specific, particularly adult-focused, genetic research. Hence, we highlight a need for targeted, population-specific studies in the Singaporean context.

Biomarker discovery helps confirm the cause of hearing loss, reducing the need for other costly and extensive investigations. It can also help predict the severity and likely progression of the condition, inform regarding recurrence risks for family planning, and holds potential for advancing precision medicine through matching patients to optimal treatment, thereby improving patient outcomes. Different types of hearing loss-associated variants implicate appropriate disease management as well as therapeutic approach. For example, both *SLC26A4* and *GJB2-*associated hearing loss are different in their severity and progressive nature, possibly implicating the timing of rehabilitative interventions such as cochlear implants [14]. In addition, identification of a genetic cause can possibly pave the way for future gene therapies. In fact, clinical studies on gene therapy for OTOF-related hearing loss are underway and early results have shown promising outcomes with dramatic improvements in hearing in some patients [15]. Hence, data from this study can provide crucial insights for diagnosis, intervention, and genetic counselling for underrepresented patients with NSHL.

This study aims to identify genetic variants associated with NSHL in a Singaporean cohort and to evaluate molecular biomarkers for diagnosis and clinical management. By integrating variant annotation, pathogenicity assessment, and frequency data, this work seeks to expand on the understanding of hereditary hearing loss and support precision medicine initiatives in Southeast Asia.

## 2. Materials and Methods

### 2.1. Participants

#### 2.1.1. Eligibility Criteria

Eligible participants included patients with NSHL in Singapore and their immediate family members (parents or siblings) who carried mutations in genes associated with hearing impairment. The inclusion of family members enables the study of inheritance modes of identified genes, and facilitates phase resolution of potential compound heterozygous variants.

#### 2.1.2. Screening Process

All potential participants underwent standardized screening using a structured questionnaire. Information collected included demographic data (age, sex, ethnicity), perinatal history, age of onset, laterality and symmetry of impairment, and type of loss (sensorineural or conductive). To prioritize cases with likely genetic causes, individuals with a history of syndromic, conductive, or non-genetic prenatal/perinatal hearing impairment risk factors (such as intrauterine infections or specific medication exposures) were excluded. The criteria further excluded those with acquired hearing issues like acoustic neuroma, head trauma, meningitis, Meniere’s disease, otitis media, ototoxic medication exposure, noise exposure, and viral or bacterial labyrinthitis.

#### 2.1.3. Ethical Considerations

Written informed consent was obtained from all patients and their participating family members. The study was approved by the SingHealth Centralised Institutional Review Board under approval protocol 2019-2337-CIRB on 2 July 2019.

In overall, a total of 115 individuals (probands) with varying levels of NSHL from mild, moderate, severe to profound was recruited. Participants’ age ranged 16–81 years and comprised all major ethnicity in Singapore population, including 88.7% Chinese, 6.1% Malay, 3.5% Indian and 1.7% other Southeast Asian. This ensured the study reflects the nation’s ethnically diverse population to the best it could.

### 2.2. Sample Preparation and Sequencing Workflow

Peripheral blood collection was carried out utilising 5 mL EDTA tubes. The DNA extraction and purification were performed efficiently using Qiagen’s QIAmp DNA Blood Mini Kit (Qiagen, Hilden, Germany). A quality check for the purity of the extracted DNA (between AD260/AD280 of 1.8 to 2.0) was performed before proceeding with sequencing steps.

#### Whole-Exome Sequencing

Library preparation was performed using the Agilent SureSelectXT Human All Exon V6 kit (Agilent Technologies, Santa Clara, CA, USA) following the manufacturer’s instructions. In brief, genomic DNA was randomly sheared into short fragments with the size of 180–280 bp. Subsequently, fragments were end repaired, A-tailed, and further ligated with the adapters. Adapter-ligated fragments were PCR-amplified, size-selected, and purified. The prepared libraries were hybridized with biotin-labeled probes, and streptavidin-coated magnetic beads were used to capture targeted exons. After washing away non-hybridized fragments and digesting the probes, the captured libraries were further enriched by PCR amplification.

The library was checked with Qubit and real-time PCR for quantification and bioanalyzer for size distribution detection. Quantified libraries were pooled and then paired-end sequenced using a NovaSeq 6000 sequencing system (Illumina, San Diego, CA, USA).

### 2.3. Bioinformatics Pipeline

Sequencing reads were aligned to the GRCh38/hg38 human reference genome using BWA (Burrows–Wheeler Aligner v0.7.17). The resulting SAM files were converted to BAM format, sorted, and indexed using SAMtools. Duplicate reads were removed and base quality score recalibration was performed with the Genome Analysis Toolkit (GATK, Broad Institute) prior to variant calling as well as to improve variant calling accuracy. Variants were then identified with GATK’s HaplotypeCaller, focusing on single-nucleotide variants (SNVs) and small insertions/deletions (indels). Stringent quality control filters were applied to exclude low-confidence calls. Only variants meeting established thresholds for read depth (≥20), mapping quality (≥30), and base quality (≥30) were retained for further analysis. Variants passing quality filters were annotated using ANNOVAR (v2020Jun07) [16] to obtain functional information including predicted coding effect (e.g., synonymous/nonsynonymous, stop-gain etc.), and integrated population allele frequencies from public databases such as gnomAD, ExAC and the 1000Genomes Project. Additional annotation included gene-based information, regulatory region mapping, and conservation scores, enabling comprehensive characterization and prioritization of variants with potential clinical relevance.

Variants were first filtered by hearing loss-associated genes recorded in the OMIM (Online Mendelian Inheritance in Man) [17] database. Then, variants with a minor allele frequency (MAF) ≤ 1% in any population database were retained. The clinical significance of the remaining variants was interpreted using ClinVar [18], dbSNP [19], gnomAD [20] and Deafness Variation Databases [21,22], following the classification ACMG-AMP 2015 guidelines of “Pathogenic”, “Likely pathogenic”, “Benign”, “Likely benign” and “Uncertain significance”.

To assess potential pathogenicity, amino acid substitutions were further evaluated using multiple in silico prediction tools. PROVEAN and SIFT predicted the effect of missense variants on protein function based on sequence homology and amino acid properties. PolyPhen-2 estimated the likelihood of structural or functional disruption of the encoded protein, and CADD (Combined Annotation Dependent Depletion) provided an integrative score reflecting the deleteriousness of each variant across multiple annotations. Through this method, potentially pathogenic variants whose allele frequency was low and which in silico prediction pointed towards being potentially deleterious were also identified.

### 2.4. Variant Validation Using Sanger Sequencing

Disease-causing variants identified through next-generation sequencing (NGS) were confirmed by Sanger sequencing. Briefly, regions of interest were amplified using polymerase chain reaction (PCR) to generate specific DNA fragments. The PCR products were then purified and subjected to Sanger sequencing to verify the presence, zygosity, and accuracy of the identified variants. This confirmation step ensured that only validated genetic variants were reported, minimizing the risk of false positives from high-throughput sequencing.

**Figure 1:**
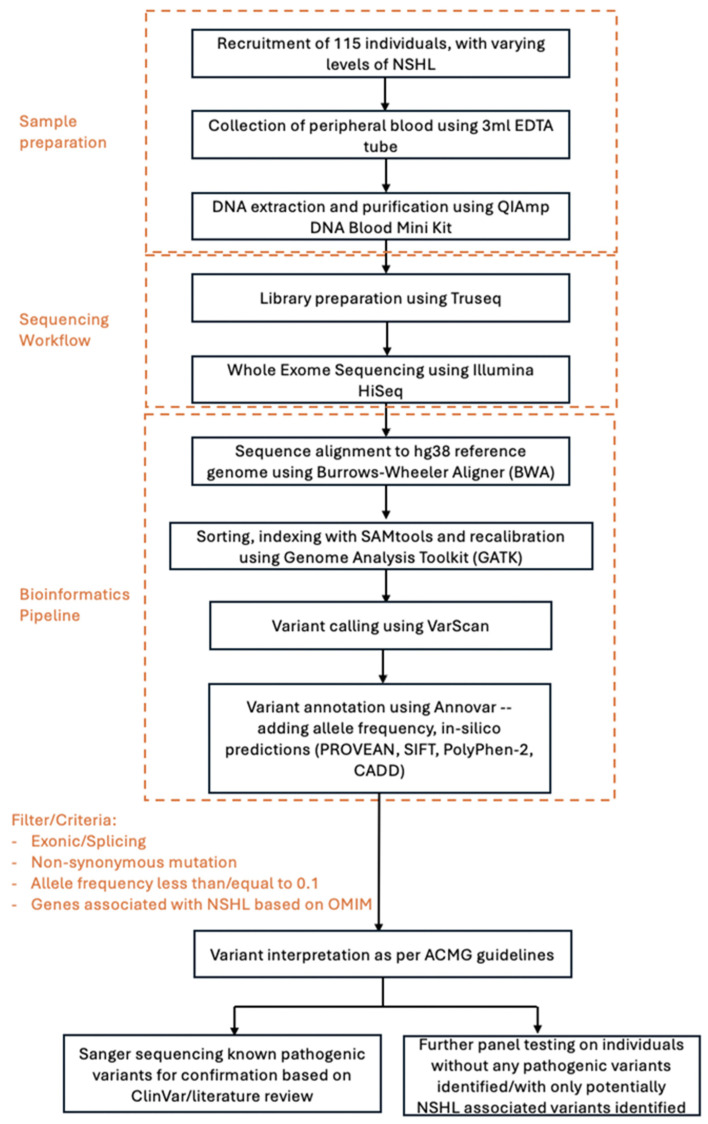
Overview of methodology. Flow diagram outlining the workflow of sample preparation, sequencing, bioinformatics pipeline and analysis.

The workflow of the methodology, spanning from sample recruitment to variant validation, is presented in Figure 1.

## 3. Results

### 3.1. Summary of Demographic and Clinical Characteristic of NSHL Patients

A total of 115 probands were included in the study. Of these, 56.5% were male, and all major ethnic groups in the Singaporean population were included. The patients’ ethnicity percentages were 88.7% Chinese, 6.1% Malay, 3.5% Indian and 1.7% other Southeast Asian (Indonesian and Filipino). Participants ranged in age from 16 to 81 years (age of onset/at diagnosis from newborn to 76 years old). Overall, 37.4% experienced onset of hearing loss before the age of 20, including 17.4% with congenital onset and 10.4% with prelingual onset. In contrast, only 7.0% reported onset after the age of 60.

Regarding laterality, majority of participants had bilateral hearing loss (88.7%). The predominant audiometric configuration was down-sloping (62.6%). In terms of severity, 51.3% had worse than moderatesevere hearing loss, while the remaining 48.7 near-normal to moderate hearing loss(Table 1). All hearing loss was sensorineural in nature.

### 3.2. Overall Diagnostic Yield

Of the 115 samples analyzed, around 57% (66/115) were found to harbor causative pathogenic variants, likely pathogenic variants, or variants with high potential pathogenicity. Within this subset of diagnosed patients, the majority, around 76% (50/66), carried causative pathogenic or likely pathogenic variants, while approximately 24% (16/66) exhibited variants classified as having high potential for pathogenicity. Notably, almost two-thirds (41/66) of the diagnosed samples underwent orthogonal validation by Sanger sequencing, confirming the accuracy of the variant calls.

This distribution underscores the diagnostic yield of the WES pipeline, demonstrating that a meaningful proportion of clinically relevant variants can be identified and validated with high confidence.

### 3.3. Genomic Biomarkers Analysis and Identification

**Figure 2:**
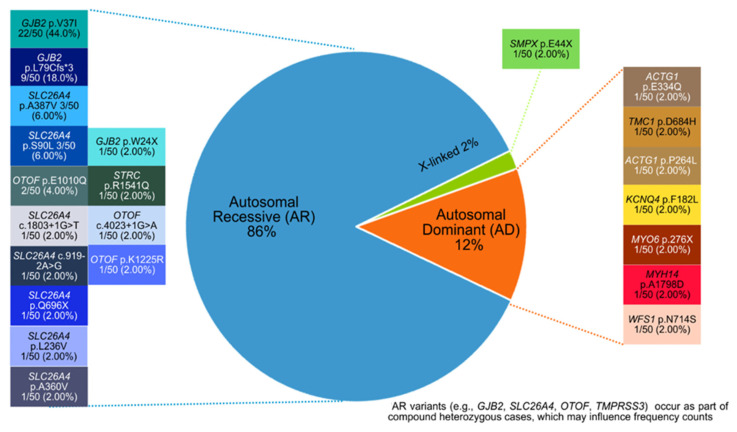
Distribution of documented pathogenic genomic biomarkers across different inheritance modes in the Singaporean cohorts.

In our cohort with documented pathogenic mutations, 86% of patients were from families with autosomal recessive inheritance, 12% from those with autosomal dominant inheritance, and 2% from those with X-linked inheritance, as shown in Figure 2. *GJB2* emerged as the most frequently mutated gene in our cohort (27.8%, 32/115), with the p.V37I and p. L79Cfs*3 variants identified in 31 of 66 diagnosed patients (47%) (Figure 2 and Supplementary Table S1). Most *GJB2* variants occurred in the homozygous state; however, four patients carried both variants as compound heterozygotes. The second most frequently mutated gene was *SLC26A4*, detected in approximately 20% (13 of 66) patients with identified causative mutations, where a diverse spectrum of variants was observed (c.1803+1G>T, c.919-2A>G, p.A360V, p.A387V, p.S90L, p.L236V). In addition, several autosomal dominant variants were identified in gene variants (*ACTG1*, *KCQN4*, *MYO6*, *TMC1*, *MYH14*), while autosomal recessive variants were identified in genes (*OTOF*, *STRC*, *TMPRSS3*). An X-linked variant in *SMPX* was also detected.

Autosomal dominant biomarkers are shown in orange, autosomal recessive biomarkers in blue, and X-linked biomarkers in green. Causative variants corresponding to each inheritance category are displayed in text boxes adjacent to their respective regions, with each text box shaded in a color matching or closely approximating the category color.

Overall, we identified three novel mutations and fourteen very rare, damaging mutations (Table 2). 70% of these highly potential pathogenic variants followed an autosomal dominant inheritance mode, including *CEACAM16*, *COL11A2*, *DIAPH1*, *MYH14*, *MYO6*, *MYO7A*, *SLC17A8*, *TECTA*, *TJP2* and *TNC*, while only 24% followed an autosomal recessive inheritance mode, including CDH23, *MYO15A* and *TJP2*. Interestingly, 3 of the variants identified were novel variants, including *TNC p.750Y*, *CDH23 p.N2063K* and *MYO6 c.554-2A>G.* For *MYO6 c.554-2A>G*, a nearby mutation, *MYO6 c.554-4A>G* was recently confirmed to be associated with hearing loss, strongly supporting the potential pathogenicity of the *MYO6 c.554-2A>G* variant [23]. Most of the genes encode stereocilia-associated proteins (e.g., *CDH23 p.N2063K*, *CDH23 p.Q2227P* [24], *DIAPH1 p.R243W* [25], *MYH14 p.A1798D*, *MYH14 p.D1023Y*, *MYH14 p.R1777C* [26], *MYO6 c.554-2A>G* [23], *MYO15A c.4143-1G>A*, *MYO15A p.G2177R* [27], *MYO7A p.G2177R* [28], *SLC17A8 p.G246A* [29], *TJP2 p.A116T* [30]) where disruption of their respective encoded protein function is known to affect the transduction of auditory signals and hence result in sensorineural hearing loss (SNHL).

### 3.4. Genotype and Clinical Phenotype Manifestation/Association

As shown in Table 3, within our Singaporean cohort, *GJB2* demonstrated the clearest genotype–phenotype relationship in relation to the severity of NSHL. Homozygous missense *GJB2* variants such as p.V37I were typically associated with bilateral mild to moderate SNHL and often had a later onset. This genotype–phenotype link was observed in 90% (18 out of 20) of patients carrying this *GJB2* point mutation. On the other hand, homozygous truncating *GJB2* variants, such as p. L79Cfs*3 or p.W24X, were associated with early-onset moderate to profound SNHL. This was observed in all six patients carrying the mutations.

In comparison, all eight patients with *SLC26A4* mutations had congenital or early-onset severe to profound SNHL. Most of these patients had Incomplete Partition type II (IP-II) inner ear malformation as diagnosed on computed tomography (CT) scans. The dominant genes detected, such as *ACTG1*, *TMC1*, *MYH14* and *MYO6,* were mostly found in patients with adult-onset bilateral hearing loss of varying severity (observed in four-fifths of patients). Furthermore, the X-linked *SMPX* gene detected was associated with severe to profound hearing loss detected around 5–6 years old while the recessive *STRC* gene was associated with congenital moderate SNHL. However, due to the small sample size for these biomarkers, further validation for the phenotypic correlation is required.

### 3.5. Similarities and Differences to Southeast Asia, China Regions and USA

In comparison to regional studies, including Thailand, Indonesia, USA and parts of China, similar and different genes were detected across all five regions (Table 4). Among shared biomarkers, *GJB2* and *SLC26A4* were detected across five countries. In addition, other genes like *ACTG1*, *OTOF* and *STRC* could be noted as detected in at least one other country as well, supporting regional consistency in major genes implicated in NSHL across East and Southeast Asian populations. Reports from heterogeneous populations such as the USA further support their broader clinical relevance [31,32,33,34,35,36].

## 4. Discussion

This study, the first comprehensive genetics investigation of non-syndromic hearing loss in Singaporean cohort, highlights the value of key molecular biomarkers such as *GJB2*, *SLC26A4*, and *OTOF*, which were consistently detected at high frequencies across the East Asian region. Notably, the recurrent identification of variants as *GJB2 c.235delc* and *p.V37I*, previously recognised as East Asian founder alleles [37], demonstrates their broader applicability within Singaporean individuals and reinforces their relevance in local diagnostic efforts.

The *GJB2* gene codes gap junction protein beta 2, more commonly known as connexin 26. Connexin proteins form channels called gap junctions that allow the movement of potassium ions between cells. Hearing requires the conversion of sound to electrical impulses, which involves ionic movement between cells. The high frequency and consistent detection of these variants strongly supports the incorporation of focused genetic panels as a first-tier diagnostic tool, enabling earlier, more accurate identification and reducing the diagnostic burden for affected individuals.

Additionally, in our cohort, 90% of homozygous missense *GJB2* p.V37I were observed as late-onset and associated with mild to moderate SNHL; while homozygous truncating *GJB2* variants, p. L79Cfs*3 or p.W24X, were linked with moderate to profound phenotypes that are early onset.

These findings underscore the importance and clinical utility of genetic testing not only in juvenile but also in adult hearing loss patients, supporting accurate diagnosis, guiding clinical follow-up and prognostic assessment, as well as provide crucial information for family planning.

Our study expands the spectrum of causative genes for NSHL, reporting three novel mutations and fourteen very rare damaging mutations. Among the genes identified as high potential pathogenicity, most have not been previously confirmed as causative in the context of NSHL, except for *MYH14*. This highlights a crucial gap in clinical knowledge about these genes, particularly within our Singaporean cohort, and emphasizes the need for further investigation. Our approach, which integrates allele frequency analysis, in silico pathogenicity prediction, and genomic context including conservation, proximity to known pathogenic residues, and gene-specific mechanisms, allowed us to identify variants that extend beyond those with established clinical significance. By focusing on variants with an allele frequency of less than 0.03, we ensured that pathogenic variants like *GJB2* were preserved while minimizing the inclusion of common, tolerated variants. Furthermore, SIFT as well as PolyPhen predictions, alongside CADD scores above 12, provide strong support for the potential deleterious nature of the identified variants. We also examined whether the affected sites are conserved or if nearby mutations with known pathogenicity could be used as reference points.

**Figure 3:**
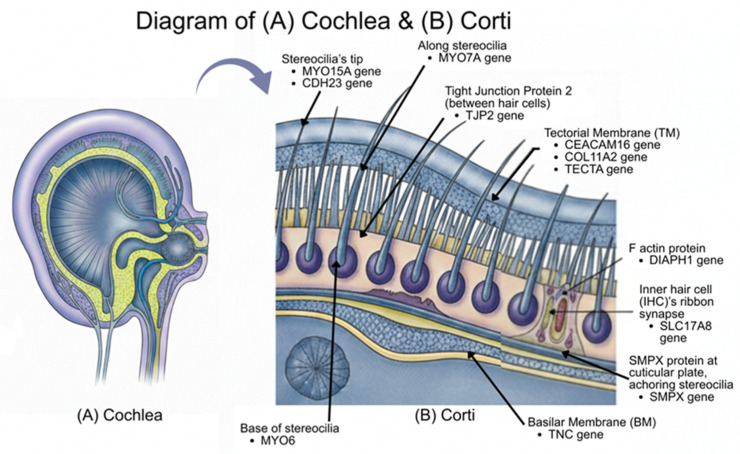
Mutation impact on the development of (**A**) cochlea and (**B**) cross-section of the corti, labelled with the protein or structure implicated by the identified potentially pathogenic variants, showing association with the inner ear.

In addition, we confirmed that all the shortlisted genes encode proteins that are involved in cochlear physiology or play a role in the noise signal transduction pathway in the inner ear, reinforcing their relevance in NSHL (Figure 3). Notably, the *MYO15A* exon9: c.4143-1G>A splice site variant stands out as a highly supported pathogenic variant, as canonical ±1 or 2 splice site mutations often lead to exon skipping or intronic material inclusion. Such variants, especially when previously reported in the same exon, typically result in pathogenic outcomes, particularly given the gene’s established role in hearing mechanisms [38]. Similarly, variants like *CEACAM16* p.V338L, *COL11A2* p.G353W, and *TECTA* p.H1400Y are associated with the tectorial membrane (TM), which is crucial for hearing. *CEACAM16* plays a key role in TM integrity and its adhesion to stereocilia for mechanical amplification [39], while mutations in *COL11A2* disrupt collagen protein structure, causing malformations in the TM’s ultrastructure [40]. *TECTA* encoding alpha-tectorin is critical for the normal formation of the TM, thereby affecting hearing thresholds [41] (Figure 3B). On the other hand, *TNC* encoding Tenascin-C, a glycoprotein found in the basement membrane of the basilar membrane (BM), is significant in auditory development and repair of hearing injuries [42]. These findings underscore the significance of these genes in cochlear function and support their candidacy as high potential pathogenic variants for NSHL.

Our general diagnostic yield of approximately 57% falls at the higher end of what has been reported for NSHL, where most studies typically achieve yields of 30–45% [31,43,44,45]. This elevated yield demonstrates the effectiveness of our sequencing and variant-interpretation strategy in uncovering both known and novel pathogenic variants within this cohort, highlighting the potential for improving NSHL diagnosis in populations with limited genetic data.

One limitation of our study is the overrepresentation of Chinese ethnic individuals, reflecting the national demographic distribution, while the smaller sample sizes of other ethnic groups limit ethnicity-specific comparisons and reduce the representativeness of findings for non-Chinese populations. Therefore, the analysis was conducted at the level of the Singaporean population as a whole to provide results that are more applicable at the national clinical level. In our data, 17% of cases were unknown onset, which may introduce some bias in the clinical phenotype–genotype correlation. This limitation should be considered when interpreting the results. Future studies with more precise data collection regarding the onset of disease could help clarify the impact of this variable and provide a more accurate analysis. Additionally, the lack of consanguineous marriage data is also a limitation in our study. Consanguineous marriage rates are known to be higher in certain regions, including parts of Asia, the Middle East, and Africa, which could potentially influence genetic analyses. However, in our study, the effect of consanguinity is likely minimal due to low prevalence in Singapore [46]. We acknowledge this limitation and recommend that future research should collect consanguinity data to better assess its impact on genetic analysis on NSHL. In addition, some variants were classified as of uncertain significance, and challenges in confirming the genetic basis for certain samples limited our detection rate. This reinforces the need for further investigation of variants unique to the Singaporean population to enhance diagnostic accuracy. Furthermore, environmental and epigenetic factors are likely to influence penetrance and expressivity, suggesting that further validation studies are essential to confirm genotype-phenotype associations in this cohort.

While our study contributes to an expanded framework for interpreting NSHL variants, functional validation of the identified variants is crucial for definitively establishing their pathogenicity. Furthermore, this work highlights the importance of integrating in silico prediction tools and clinical phenotypes with empirical data to support genomic interpretation, particularly in settings where reference data remain scarce.

## 5. Conclusions

In summary, our study provides important insights into the genetic architecture of NSHL within Singaporean populations. By identifying both known and novel pathogenic variants, we demonstrate an improved yield in molecular diagnosis and highlight the value of genetic testing in guiding clinical management decisions. The discovery of new mutations further broadens the diagnostic spectrum, underscoring the complexity and diversity of NSHL in the region.

Overall, these findings support the clinical utility of targeted genetic testing in NSHL and provide a framework for improved diagnostic strategies and genetic counseling tailored to the region. Furthermore, by enabling more precise molecular classification, this work contributes to the implementation of precision medicine approaches in clinical genetics and hearing loss management.

## Figures and Tables

**Table 1:** Demographic and clinical characteristics of patients with non-syndromic hearing loss.

	Total (*n* = 115)
Male gender	56.5% (65)
Ethnicity	
Chinese	88.7% (102)
Malay	6.1% (7)
Indian	3.5% (4)
Misc. Southeast Asian	1.7% (2)
Age (years) [mean (SD)]	30.6 (17.7)
16–20	37.4% (43)
21–40	30.4% (35)
41–60	25.2% (29)
61–76	7.0% (8)
Onset of hearing loss	
Congenital	17.4% (20)
Prelingual	10.4% (12)
Postlingual	29.6% (34)
Adult	25.2% (29)
Unknown	17.4% (20)
Laterality of hearing loss	
Bilateral	88.7% (102)
Unilateral	11.3% (13)
Pattern of hearing loss	
Downsloping	62.6% (72)
Flat	22.6% (26)
Upsloping	5.2% (6)
Cookie bite	9.6% (11)
Severity of hearing loss (AC PTA in affected ears)	
Near-normal (≤25 dB)	11.3% (13)
Mild (26–40 dB)	22.6% (26)
Moderate (41–55 dB)	14.8% (17)
Moderately severe (56–70 dB)	14.8% (17)
Severe (71–90 dB)	8.7% (10)
Profound (>90 dB)	27.8% (32)

Variables are expressed as *n*% or mean (SD).

**Table 2:** Identified high-potential pathogenic variants in NSHL Singaporean population.

Patient Code Number	Gene	Nucleotide Change	Protein Change	Novel?	In Silico Predictions				Allele Frequency on gnomAD, 1000 Genomes and dbSNP	
					SIFT	POLY	CADD	REVEL	Global	East Asian
Patient number 49	*CDH23*	c.T6189A	p.N2063K	Yes	D	D,D	13.6	NA	.	.
Patient number 49	*CDH23*	c.A6680C	p.Q2227P	No	T	P,D	17.7	0.208	7.00 × 10^−6^	0 to 0.0002
Patient number 96	*CEACAM16*	c.G1012T	p.V338L	No	D	D,D	25.5	0.125	0 to 0.0003	0 to 0.0017
Patient number 102	*COL11A2*	c.G1057T	p.G353W	No	D	D,D	28.6	0.531	0.000080 to 0.0002	0.0010 to 0.0023
Patient number 33	*DIAPH1*	c.C127T	p.R243W	No	D	D,D	32	0.517	0.000007 to 0.00006	0
Patient number 45	*MYH14*	c.C5393A	p.A1798D	No	D	P,P	26.3	0.343	0.000027 to 0.00047	0.00 to 0.0008
Patient number 22	*MYH14*	c.G3067T	p.D1023Y	No	D	P,P	27.9	0.553	6.198 × 10^−7^	0.000025
Patient number 16	*MYH14*	c.C5329T	p.R1777C	No	D	D,D	27.5	0.608	0.00014 to 0.0010	0.000 to 0.0020
Patient number 21	*MYO15A*	c.4143-1G>A	.	No	NA	NA	25.3	NA	7 × 10^−6^ to 0.0002	0.0002–0.0010
Patient number 50	*MYO15A*	c.C214T	p.R72C	No	D	D,D	32	0.415	6.201 × 10^−7^	0
Patient number 62	*MYO7A*	c.G6529A	p.G2177R	No	D	D,D	32	0.751	2.9 × 10^−6^	0
Patient number 79	*SLC17A8*	c.G737C	p.G246A	No	D	D,D	29.7	0.794	0 to 0.0002	0 to 0.0009
Patient number 43	*SMPX*	c.A55G	p.N19D	No	D	D,D	25.3	0.322	0 to 0.000055	0 to 0.0017
Patient number 25	*TECTA*	c.C4198T	p.H1400Y	No	T	D,P	25.4	0.426	0 to 0.0002	0 to 0.0015
Patient number 04	*TJP2*	c.G346A	p.A116T	No	D	D,D	23.5	NA	0.000174 to 0.0006	0.0026 to 0.0069
Patient number 35	*TNC*	C.A2248T	p.N750Y	Yes	D	D,D	28.2	NA	.	.
Patient number 17	*MYO6*	c.554-2A>G	NA	Yes	NA	NA	NA	NA	.	.

Supporting information includes in silico prediction pointing towards deleterious/damaging and low allele frequency. D: Damaging; P: Probable Damaging. NA: Not Available.

**Table 3:** List of causative variants and correlated phenotype presentation in Singaporean population.

Inheritance Mode	Variant	Patient Code Number	Any Other Variants Carried (Heterozygous)	Unilateral or Bilateral	Hearing Profile	Age of Onset ^#^
Autosomal dominant	*ACTG1 p:P264L*	Patient Number 03	No	Bilateral	Down-sloping moderate to profound SNHL	Left ear: 35 years old, Right ear: 40 years old
	*ACTG1 p.E334Q*	Patient Number 50	No	Bilateral	Moderate SNHL	~Late 30 s
	*MYH14 p.A1798D*	Patient Number 45	No	Bilateral	Severe to profound SNHL	21 years old **
	*MYO6 p.R276X*	Patient Number 46	Carrier of heterozygous GJB2 p.V37I	Bilateral	Mild SNHL	23 years old
	*TMC1 p.D684H*	Patient Number 28	No	Unilateral	Profound SNHL	10 years old
	*WFS1 p.N714S*	Patient Number 73	No	Bilateral	Mild to moderate SNHL	8 years old
Autosomal recessive (homozygous)	GJB2 p.L79Cfs*3	Patient Number 14	No	Bilateral	Severe to profound SNHL	Congenital
		Patient Number 15			Severe to profound SNHL	Congenital
		Patient number 96			Moderate to severe SNHL	3 years old
		Patient number 97			Moderate to severe SNHL	Congenital
		Patient number 104			Severe to profound SNHL	Congenital
	*GJB2 p.V37I*	Patient number 09	No	Bilateral	Severe to profound SNHL	22 years old **
		Patient number 18			Moderate SNHL	9 years old
		Patient number 20			Down-sloping mild sloping to moderate SNHL	29 years old
		Patient number 24			Moderate high-frequency SNHL	19 years old (incidental finding, asymptomatic)
		Patient number 34			Mild to severe sloping SNHL	Early 40s
		Patient number 42			Mild to moderate SNHL	13 years old
		Patient number 51			Mild to moderate SNHL	6 years old
		Patient number 54			Moderate to severe SNHL	67 years old **
		Patient number 55			Mild SNHL	7 years old
		Patient number 60			Mild to moderate SNHL	14 years old
		Patient number 66			Mild to moderate SNHL	45 years old
		Patient number 68			Mild SNHL	14 years old
		Patient number 70			Mild SNHL	14 years old
		Patient number 71			Mild to moderate SNHL	Congenital
		Patient number 94			Mild to moderate SNHL	25 years old
		Patient number 100			Mild to moderate SNHL	6 months
		Patient number 105			Mild SNHL	26 years old
		Patient number 109			Mild to moderate SNHL	Left: 2 months, Right: 6 years old
		Patient number 113			Moderate SNHL	Congenital
		Patient number 118			Mild to moderate SNHL	Congenital
	*GJB2 p.W24X*	Patient number 82	No	Bilateral	Profound SNHL	2 years old
	*SLC26A4 p.A387V*	Patient number 80	No	Bilateral	Severe to profound	1 years old
		Patient number 84			Severe to profound SNHL	Congenital
	*SLC26A4 p.L236V*	Patient number 83	No	Bilateral	Profound SNHL	Congenital
	*STRC p.R1541Q*	Patient number 93	No	Bilateral	Moderate SNHL	Congenital
Autosomal recessive (compound heterozygous)	*GJB2 p.V37I* and GJB2 p.L79Cfs*3	Patient number 69	No	Bilateral	Mild to moderate SNHL	14 years old
		Patient number 87			Mild to moderate SNHL	25 years old **
		Patient number 89			Severe to profound SNHL	5 years old
		Patient number 115			Mild to moderate SNHL	9 years old
	*OTOF p.E1010Q* and *OTOF c.4023+1G>A*	Patient number 72	No	Bilateral	Moderate SNHL	20 years old **
	*SLC26A4 c.919-2A>G* and *c.1803+1G>T*	Patient number 05	No	Bilateral	Severe to profound	13 month
	*SLC26A4 p.A360V* and *p.A387V*	Patient number 19	No	Bilateral	SNHL, severe on L; mild–moderate–severe to profound	Congenital
	*SLC26A4 p.S90L* and *SLC26A4 c.919-2A>G*	Patient number 30	No	Bilateral	Severe to profound SNHL	Congenital
		Patient number 31			Severe to profound SNHL	Congenital
	*SLC26A4 p.S90L* and *SLC26A4 p.Q696X*	Patient number 53	No	Bilateral	Severe to profound SNHL	10 years old
	*TMPRSS3 p.L57S* and *TMPRSS3 p.R16X*	Patient number 99	No	Bilateral	Severe to profound SNHL	Congenital
	*OTOF p.E1010Q* and *OTOF p.K1225R*	Patient number 88	No	Bilateral	Moderate SNHL	18 months
Autosomal dominant and Autosomal recessive (homozygous)	*KCQN4 p.F182L* (*Het*) and *GJB2 p.V37I* (*Hom*)	Patient number 114	No	Bilateral	Mild to moderate SNHL	21 years old
X-linked	*SMPX p.E44X*	Patient number 38	No	Bilateral	Severe to profound SNHL	5–6 years old

** Confirmed age at diagnosis. ^#^ Classification of hearing loss onset is based on the age of the first diagnosis, with progressive hearing loss cases categorized according to initial diagnosis age. Hom: Homozygous, Het: Heterozygous.

**Table 4:** Relative comparison of biomarkers/genes detected as causative variants recorded in studies across nearby regions.

	Thailand	Indonesia	China	United States	Our Cohort
*ACTG1*					
*ADGRV1*					
*CDH23*					
*COCH*					
*DIAPH3*					
*GJB2* *					
*GJB3*					
*GJB6*					
*GRHL2*					
*GRXCR2*					
*GSDME*					
*KARS*					
*KCQN4*					
*LOXHD1*					
*MITF*					
*MYO15A*					
*MYO6*					
*MYO7A*					
*OSBPL2*					
*OTOF*					
*OTOG*					
*PAX3*					
*PCDH15*					
*PDZD7*					
*POU3F4*					
*PTPRQ*					
*RRM2B*					
*SALL1*					
*SLC26A4* *					
*SMPX*					
*SOX10*					
*STRC*					
*TBC1D24*					
*TECTA*					
*TIMM8A*					
*TMC1*					
*TMPRSS3*					
*USH2A*					
*WFS1*					

Red: Shared identical variants; Grey: Unique variants. * Indication of genes common across the regions.

## Data Availability

Data are available from the corresponding author upon reasonable request but are not publicly available due to clinical sensitivity and ethical restrictions.

## Supplementary Materials

The following supporting information can be downloaded at: https://www.mdpi.com/article/10.3390/biom16030352/s1, Table S1: Complete list of identified pathogenic variants; Table S2: Complete list of identified variants in the Singaporean cohort; Figure S1. Chromosome ideogram of the distribution of NSHL-associated genes based on inheritance mode [47,48]; Supplementary VCF.txt; Supplementary Data_Sanger results.

## Abbreviations

The following abbreviations are used in this manuscript:

NSHL	Nonsyndromic hearing loss
WHO	World Health Organization
NGS	Next-generation sequencing
PCR	Polymerase chain reaction
SNHL	Sensorineural hearing loss
TM	Tectorial membrane
AD	Autosomal dominant
AR	Autosomal recessive
