# Supplementary material for: Genomic Biomarkers and Mutational Landscape of Nonsyndromic Hearing Loss (NSHL) in the Singaporean Population: Clinical Translational Implications
Source: Biomolecules. 2026 Feb 26;16(3):352. doi: 10.3390/biom16030352 (PMC13024665; doi:10.3390/biom16030352)
Supplement: Supplementary file 1 [file biomolecules-16-00352-s001.zip › biomolecules-4126176-supplementary.pdf]

biomolecules

MDPI

Supplementary Materials

Table S1. Complete list of identified documented pathogenic variants.

Inheritance Mode	Gene	Nucleotide/Amino Acid Position	Frequency
AD	*ACTG1*	p.P264L	1
		p.E334Q	1
	KCNQ4	p.F182L	1
	MYH14	p.A1798D	1
	MYO6	p.R276X	1
	TMC1	p.D684H	1
	WFS1	p.N714S	1
AR	*GJB2*	p.L79Cfs*3	9
		p.V37I	22
		p.W24X	1
	OTOF	p.E1010Q	2
		p.K1225R	1
		c.4023+1G>A	1
	SLC26A4	c.1803+1G>T	1
		c.919-2A>G	3
		p.Q696X	1
		p.A360V	1
		p.A387V	3
		p.S90L	3
		p.L236V	1
	STRC	p.R1541Q	1
	TMPRSS3	p.R16X	1
		p.L57S	1
XL	*SMPX*	p.E44X	1

AD = autosomal dominant; AR = autosomal recessive; XL = X-linked.

Table S1: Complete list of identified documented pathogenic variants, including detailed information on the specific amino acid or nucleotide changes, their genomic positions, the mode of inheritance, and the number of samples in which each causative variant was detected.

Table S2. Complete list of identified variants in Singaporean cohort.

Genes	Variants	Documented-Highly Potential
ACTG1	p.P264L	documented pathogenic
	p.E334Q	documented pathogenic
KCNQ4	p.F182L	documented pathogenic
MYH14	p.A1798D	documented pathogenic
MYO6	p.R276X	documented pathogenic
TMC1	p.D684H	documented pathogenic
WFS1	p.N714S	documented pathogenic
GJB2	p.L79Cfs*3	documented pathogenic
	p.V37I	documented pathogenic
	p.W24X	documented pathogenic
OTOF	p.E1010Q	documented pathogenic
	p.K1225R	documented pathogenic
	c.4023+1G>A	documented pathogenic
SLC26A4	c.1803+1G>T	documented pathogenic
	c.919-2A>G	documented pathogenic

	p.Q696X	documented pathogenic
	p.A360V	documented pathogenic
	p.A387V	documented pathogenic
	p.S90L	documented pathogenic
	p.L236V	documented pathogenic
STRC	p.R1541Q	documented pathogenic
TMPRSS3	p.R16X	documented pathogenic
	p.L57S	documented pathogenic
SMPX	p.E44X	documented pathogenic
CDH23	c.T6189A	Highly Potential Pathogenic
CDH23	c.A6680C	Highly Potential Pathogenic
CEACAM16	c.G1012T	Highly Potential Pathogenic
COL11A2	c.G1057T	Highly Potential Pathogenic
DIAPH1	c.C127T	Highly Potential Pathogenic
MYH14	c.C5393A	Highly Potential Pathogenic
MYH14	c.G3067T	Highly Potential Pathogenic
MYH14	c.C5329T	Highly Potential Pathogenic
MYO15A	c.4143-1G>A	Highly Potential Pathogenic
MYO15A	c.C214T	Highly Potential Pathogenic
MYO7A	c.G6529A	Highly Potential Pathogenic
SLC17A8	c.G737C	Highly Potential Pathogenic
SMPX	c.A55G	Highly Potential Pathogenic
TECTA	c.C4198T	Highly Potential Pathogenic
TJP2	c.G346A	Highly Potential Pathogenic
TNC	C.A2248T	Highly Potential Pathogenic
MYO6	c.554-2A>G	Highly Potential Pathogenic

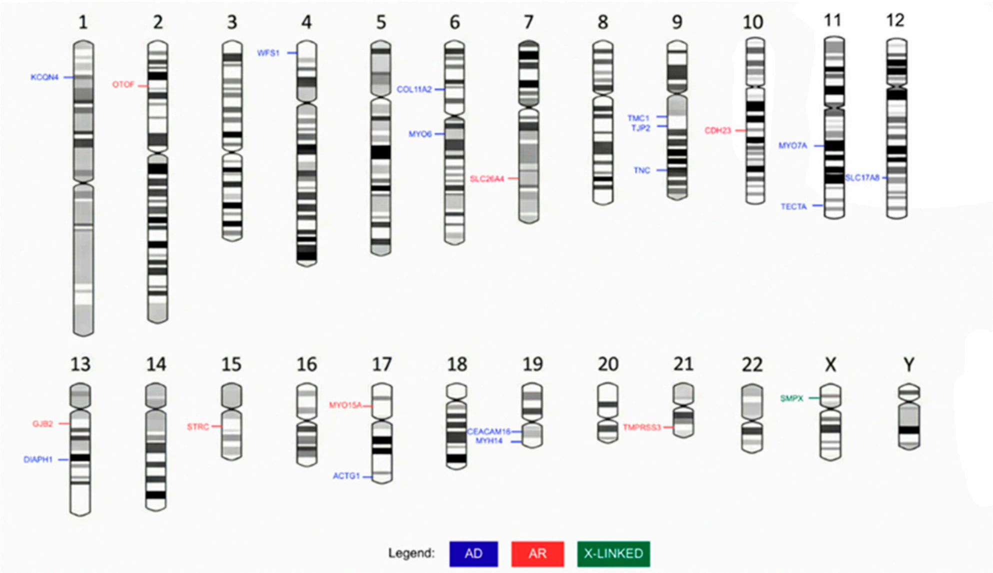

Figure S1. Chromosome ideogram of the distribution of NSHL-associated genes based on inheritance mode. Chromosome ideograms were generated using the Phenogram software [1]. Coloured regions indicated the cytogenetic band on each chromosome according to the predefined setting of the Phenogram, which is based on ideogram documented in the UCSC database [2]. Relative positions of genes are labelled with color coding based on inheritance mode. AD: Blue; AR: Red; X-linked: Green. These NSHL-associated genes (including pathogenic, likely pathogenic and high potential for pathogenicity genes) are located on chromosomes 1, 2, 4, 6, 7, 8, 9, 10, 11, 12, 13, 14, 15, 16, 17, 19, 21 and X. This reveals that NSHL-associated genes are widely distributed across the genome. Moreover, several chromosomes carry more than one gene implicated in hearing loss, such as COL11A2 and MYO6 on chromosome 6; TMC1, TJP2, and TNC on chromosome 9; MYO7A and

TECTA on chromosome 11; GJB2 and DIAPH1 on chromosome 13; MYO15A and ACTG1 on chromosome 17; and CEA-CAM16 and MYH14 on chromosome 19.

Reference:

1. Wolfe, D., et al., *Visualizing genomic information across chromosomes with PhenoGram.* BioData Min, 2013. **6**(1): p. 18.
2. Furey, T.S. and D. Haussler, *Integration of the cytogenetic map with the draft human genome sequence.* Hum Mol Genet, 2003. **12**(9): p. 1037-44.

https://doi.org/10.3390/xxxxx
