# Supplementary material for: Genomic Biomarkers and Mutational Landscape of Nonsyndromic Hearing Loss (NSHL) in the Singaporean Population: Clinical Translational Implications
Source: Biomolecules. 2026 Feb 26;16(3):352. doi: 10.3390/biom16030352 (PMC13024665; doi:10.3390/biom16030352)
Supplement: Supplementary file 1 [file biomolecules-16-00352-s001.zip › Supplementary_Data_Sanger_results.pptx]

## Slide 1
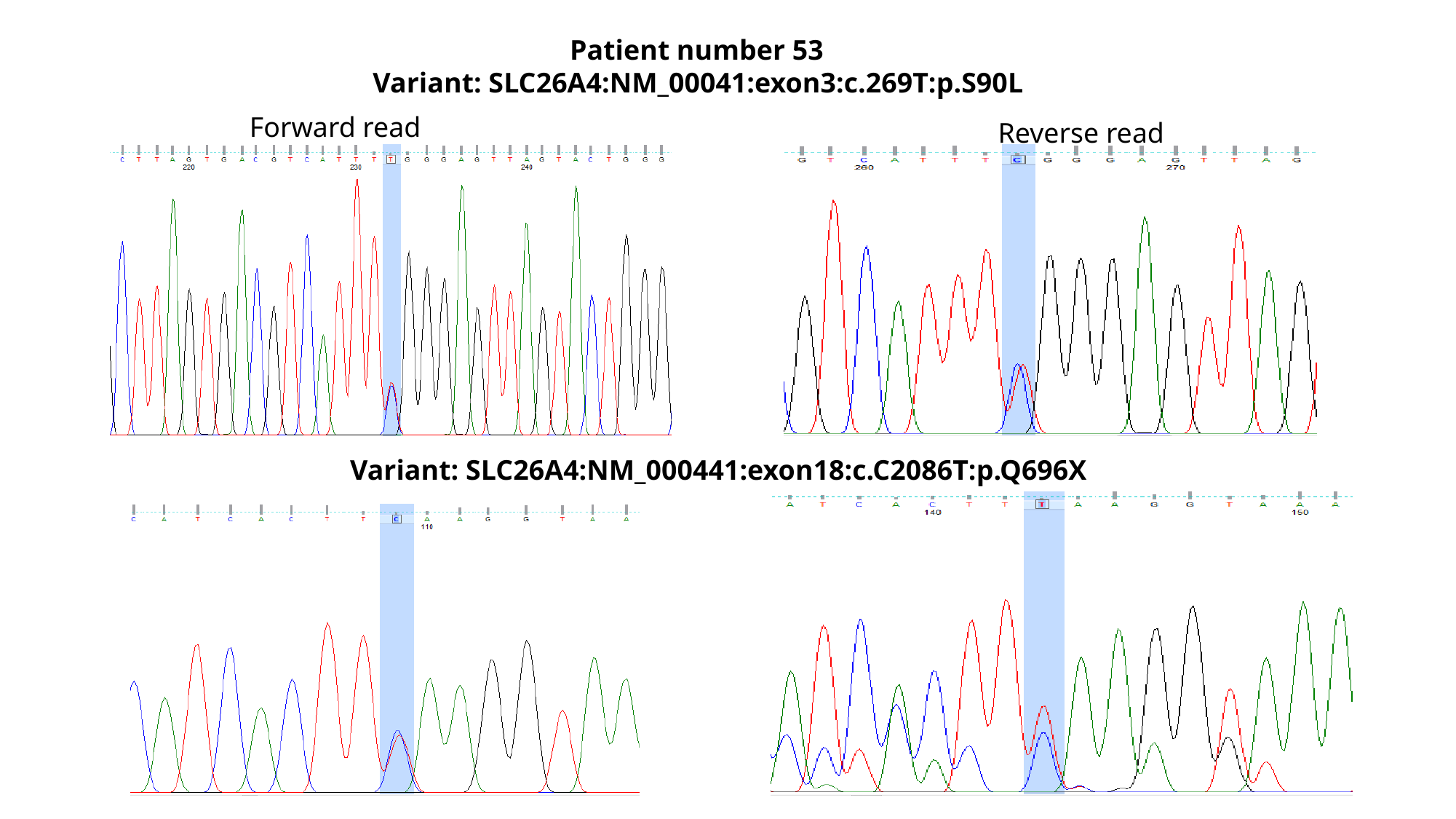

Patient number 53
Variant: SLC26A4:NM_00041:exon3:c.269T:p.S90L
Forward read
Reverse read
Variant: SLC26A4:NM_000441:exon18:c.C2086T:p.Q696X

## Slide 2
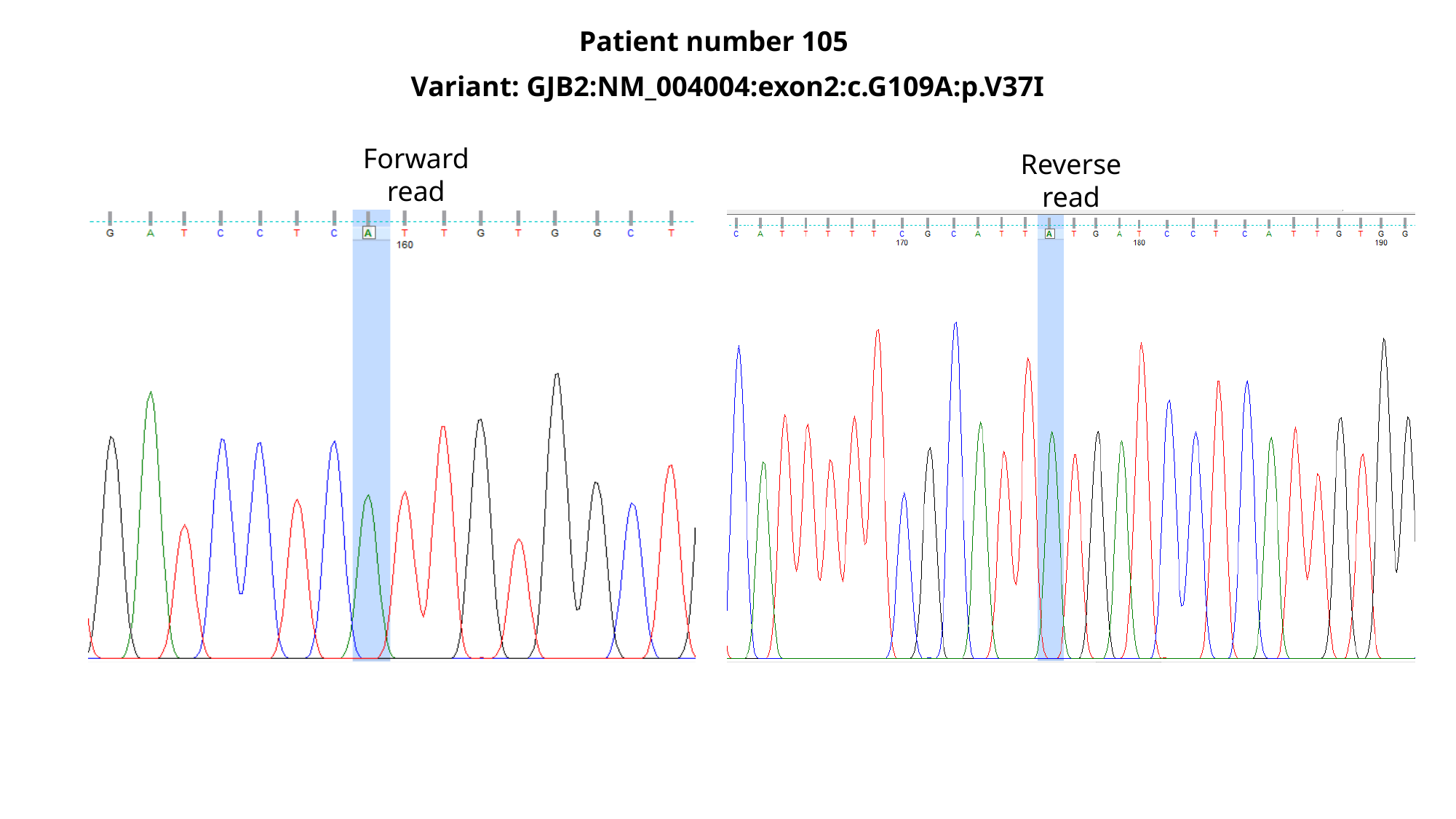

Patient number 105
Variant: GJB2:NM_004004:exon2:c.G109A:p.V37I
Forward read
Reverse read

## Slide 3
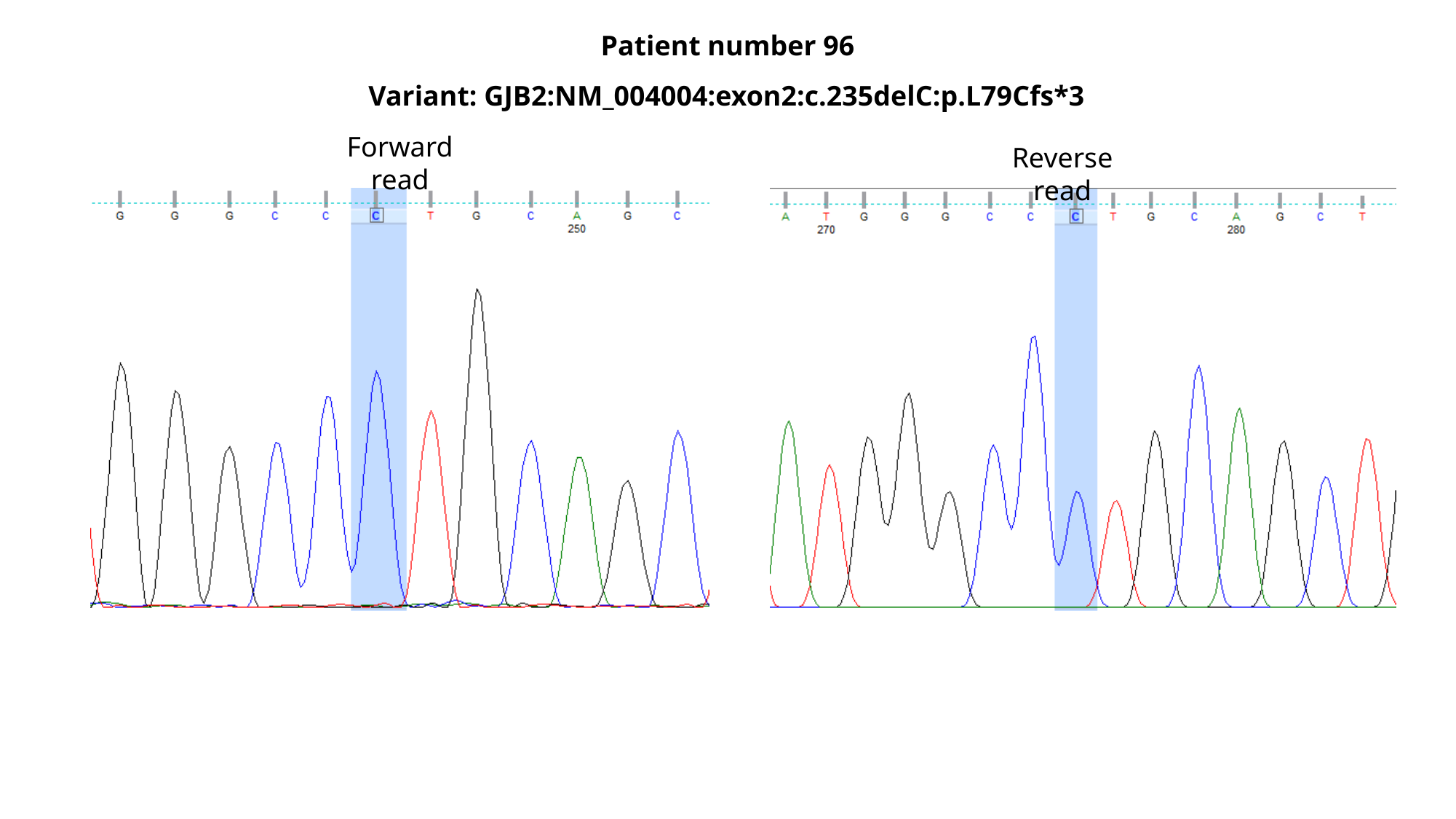

Patient number 96
Variant: GJB2:NM_004004:exon2:c.235delC:p.L79Cfs*3
Forward read
Reverse read

## Slide 4
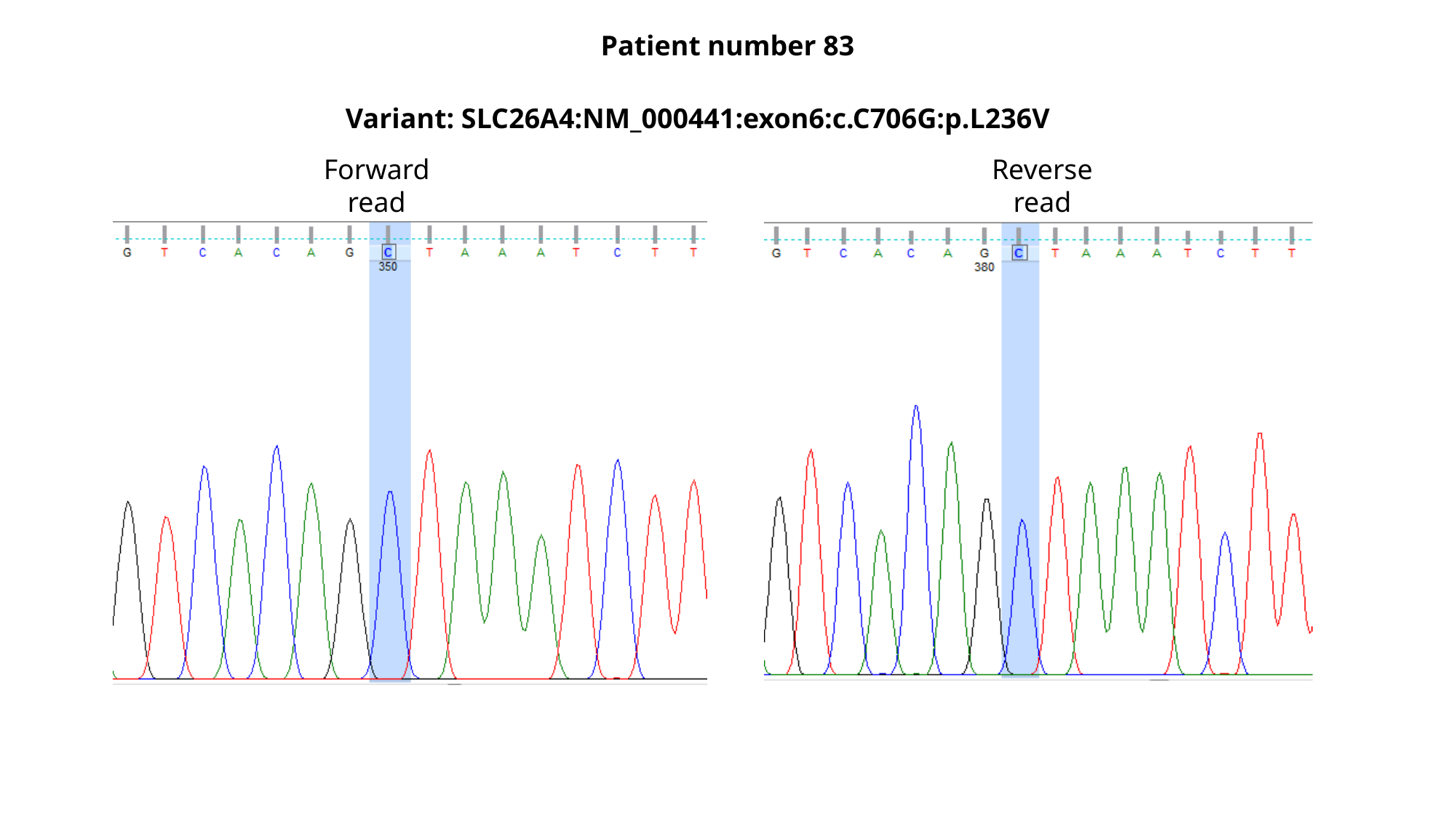

Patient number 83
Variant: SLC26A4:NM_000441:exon6:c.C706G:p.L236V
Forward read
Reverse read

## Slide 5
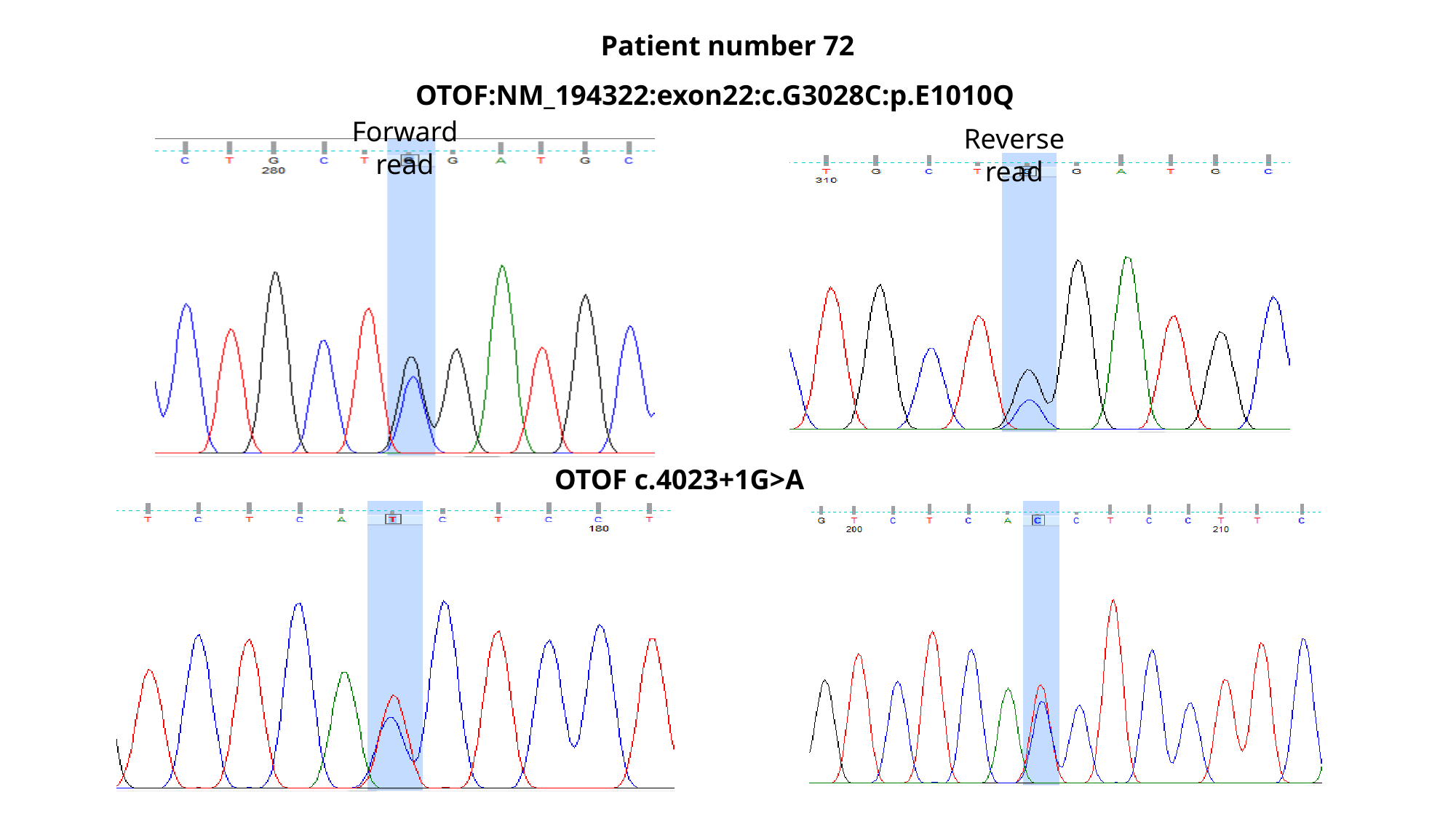

Patient number 72
OTOF:NM_194322:exon22:c.G3028C:p.E1010Q
Forward read
Reverse read
OTOF c.4023+1G>A

## Slide 6
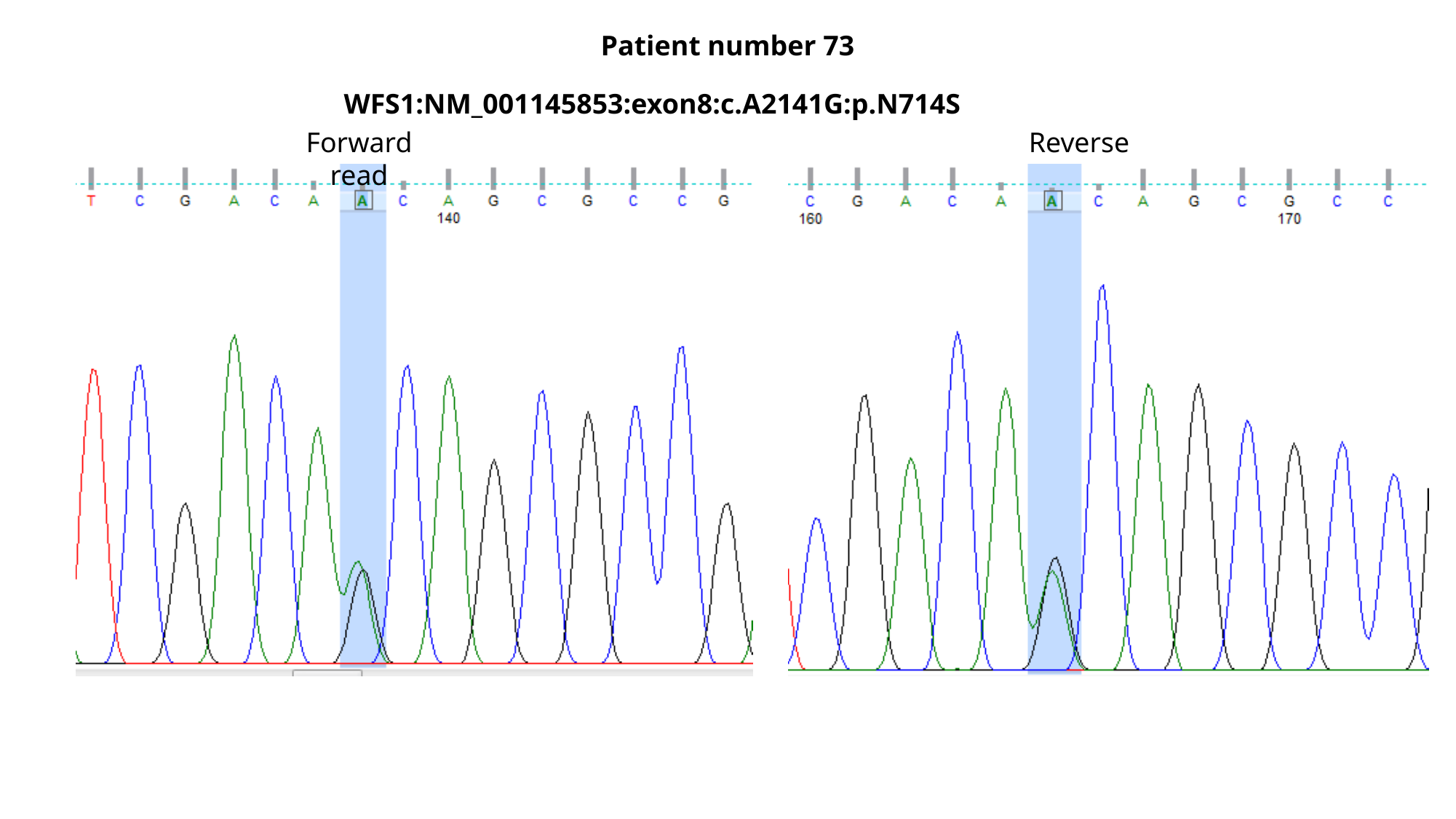

Patient number 73
WFS1:NM_001145853:exon8:c.A2141G:p.N714S
Forward read
Reverse read

## Slide 7
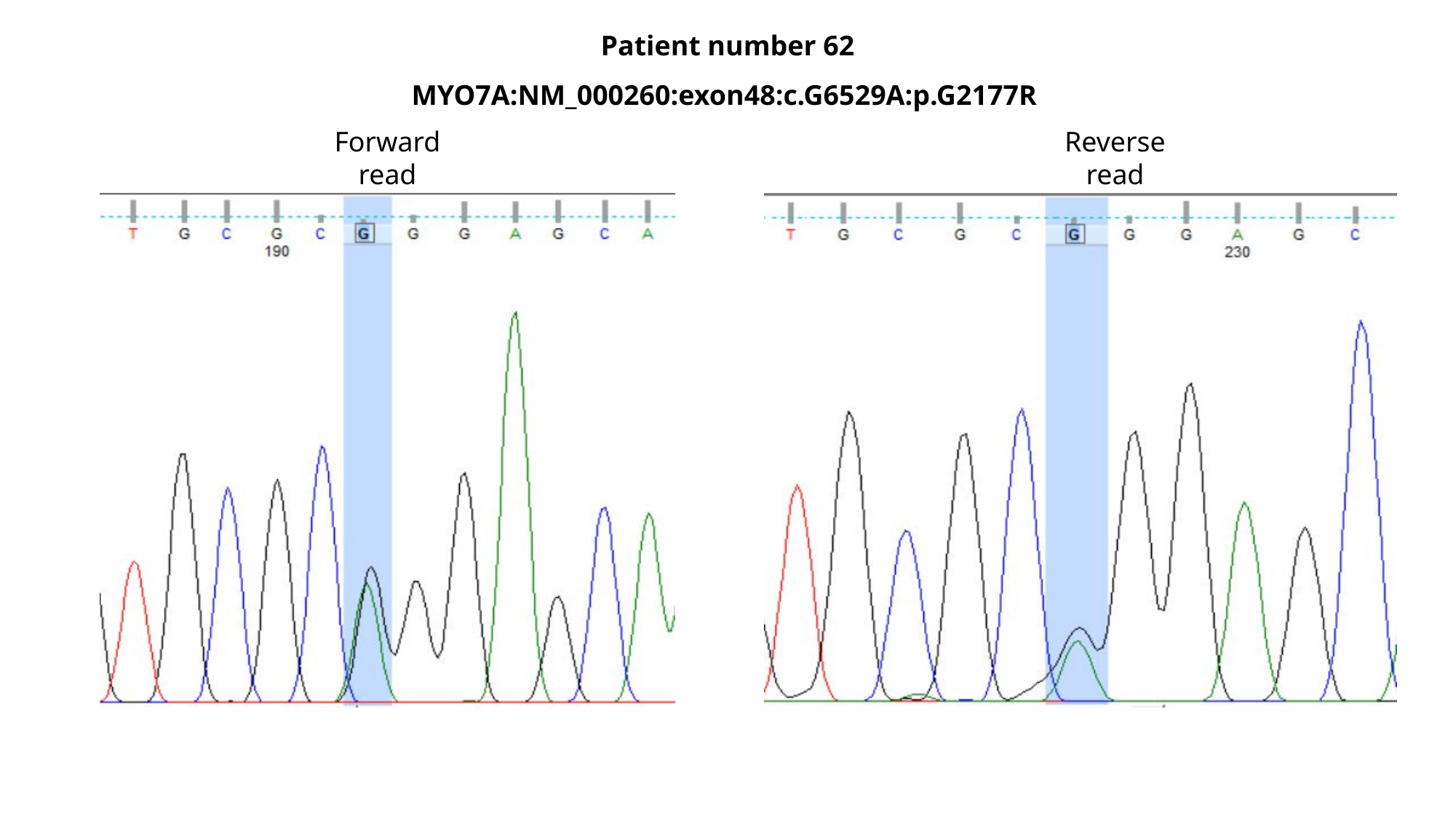

Patient number 62
MYO7A:NM_000260:exon48:c.G6529A:p.G2177R
Forward read
Reverse read

## Slide 8
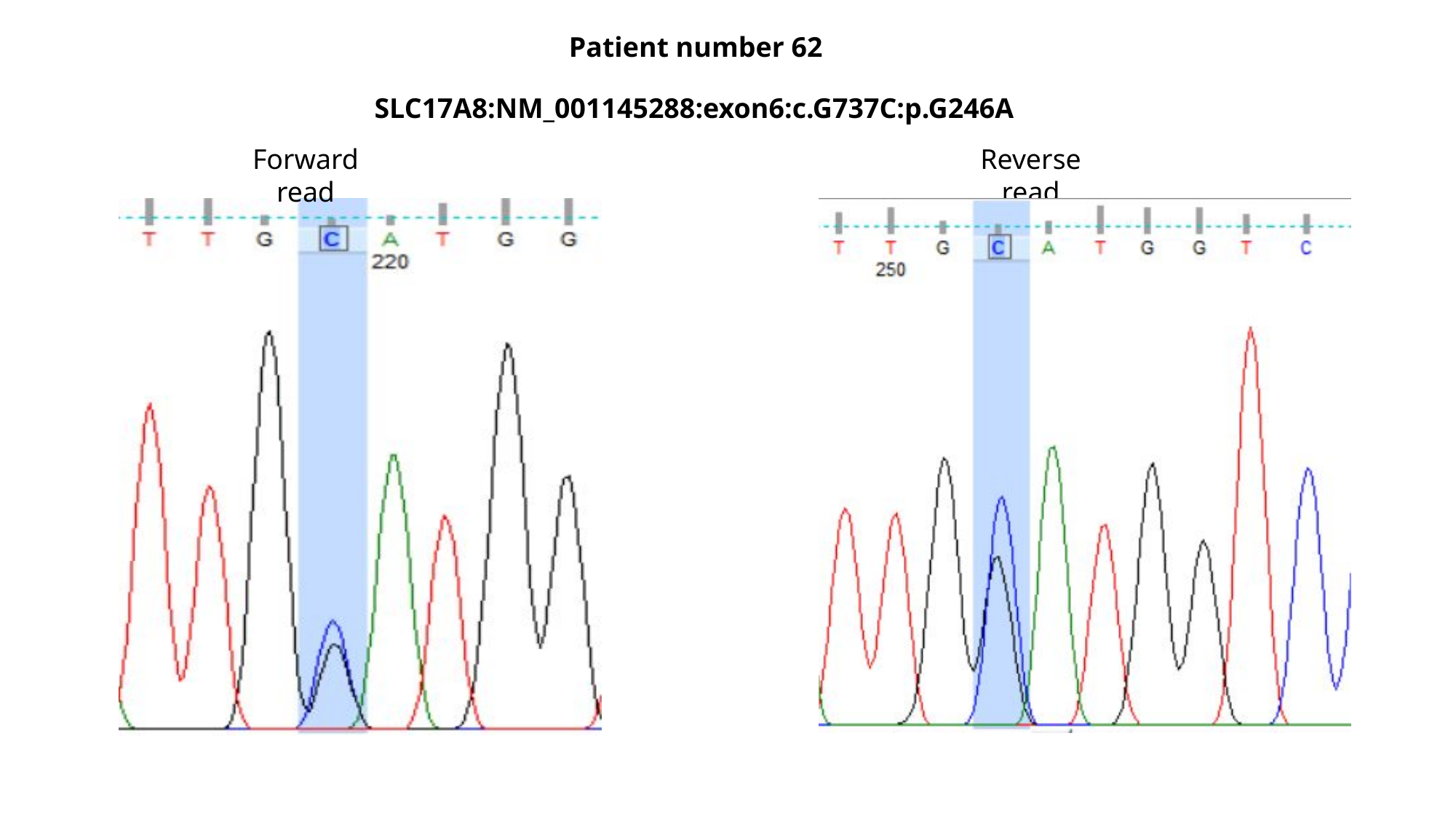

Patient number 62
SLC17A8:NM_001145288:exon6:c.G737C:p.G246A
Forward read
Reverse read

## Slide 9
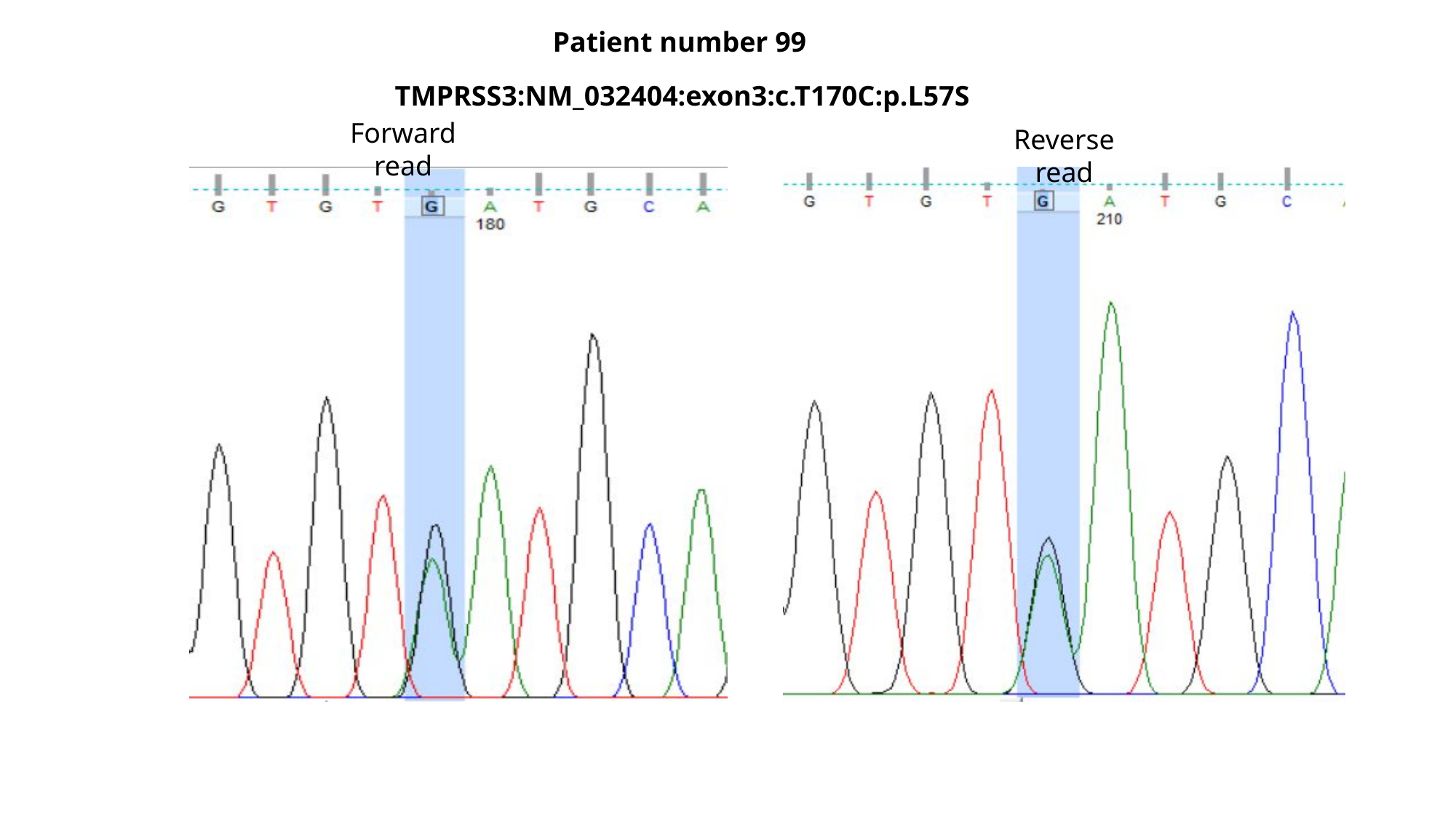

Patient number 99
TMPRSS3:NM_032404:exon3:c.T170C:p.L57S
Forward read
Reverse read

## Slide 10
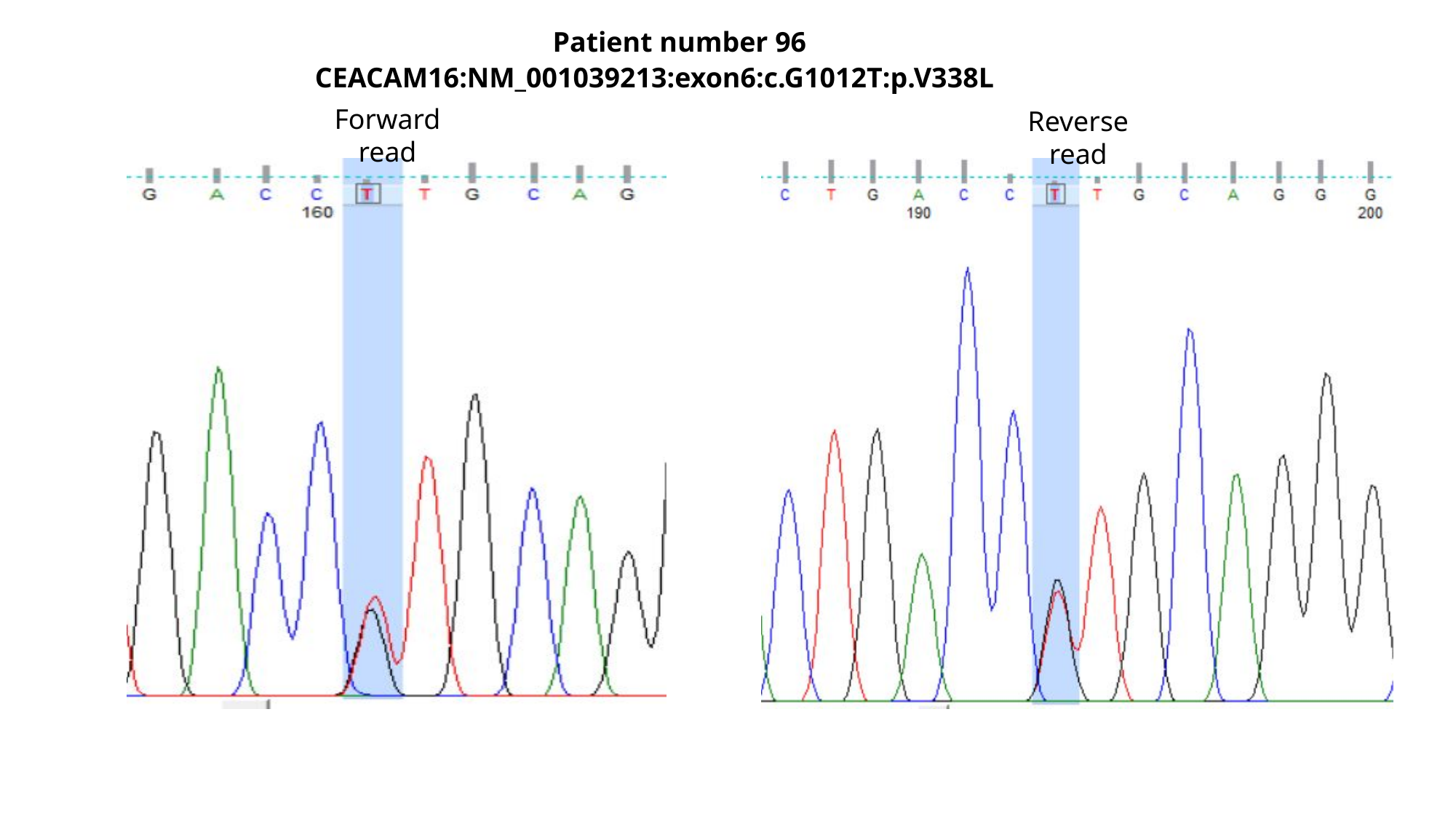

Patient number 96
CEACAM16:NM_001039213:exon6:c.G1012T:p.V338L
Forward read
Reverse read
